# Epigenetics of neural differentiation: Spotlight on enhancers

**DOI:** 10.3389/fcell.2022.1001701

**Published:** 2022-10-13

**Authors:** Mayela Giacoman-Lozano, César Meléndez-Ramírez, Emmanuel Martinez-Ledesma, Raquel Cuevas-Diaz Duran, Iván Velasco

**Affiliations:** ^1^ Tecnologico de Monterrey, Escuela de Medicina y Ciencias de la Salud, Monterrey, NL, Mexico; ^2^ Instituto de Fisiología Celular—Neurociencias, Universidad Nacional Autónoma de Mexico, Mexico City, Mexico; ^3^ Laboratorio de Reprogramación Celular, Instituto Nacional de Neurología y Neurocirugía “Manuel Velasco Suárez”, Mexico City, Mexico; ^4^ Tecnologico de Monterrey, The Institute for Obesity Research, Monterrey, NL, Mexico

**Keywords:** epigenetics, neurogenesis, enhancers, neural induction, transcription regulation, cell-type specific

## Abstract

Neural induction, both *in vivo* and *in vitro*, includes cellular and molecular changes that result in phenotypic specialization related to specific transcriptional patterns. These changes are achieved through the implementation of complex gene regulatory networks. Furthermore, these regulatory networks are influenced by epigenetic mechanisms that drive cell heterogeneity and cell-type specificity, in a controlled and complex manner. Epigenetic marks, such as DNA methylation and histone residue modifications, are highly dynamic and stage-specific during neurogenesis. Genome-wide assessment of these modifications has allowed the identification of distinct non-coding regulatory regions involved in neural cell differentiation, maturation, and plasticity. Enhancers are short DNA regulatory regions that bind transcription factors (TFs) and interact with gene promoters to increase transcriptional activity. They are of special interest in neuroscience because they are enriched in neurons and underlie the cell-type-specificity and dynamic gene expression profiles. Classification of the full epigenomic landscape of neural subtypes is important to better understand gene regulation in brain health and during diseases. Advances in novel next-generation high-throughput sequencing technologies, genome editing, Genome-wide association studies (GWAS), stem cell differentiation, and brain organoids are allowing researchers to study brain development and neurodegenerative diseases with an unprecedented resolution. Herein, we describe important epigenetic mechanisms related to neurogenesis in mammals. We focus on the potential roles of neural enhancers in neurogenesis, cell-fate commitment, and neuronal plasticity. We review recent findings on epigenetic regulatory mechanisms involved in neurogenesis and discuss how sequence variations within enhancers may be associated with genetic risk for neurological and psychiatric disorders.

## Introduction

Complex multicellular organisms are made up of trillions of highly specialized cells with distinct phenotypes and nearly identical genomes. This phenotypic heterogeneity is mainly explained by epigenetics, the field that studies mechanisms that can alter gene expression without modifying DNA sequence. Non-coding regions of the DNA, for example, enhancers, are important epigenetic mechanisms that orchestrate precise spatiotemporal gene expression patterns. Other epigenetic regulatory mechanisms include DNA methylation, histone post-transcriptional modifications, and chromatin remodeling (Yao et al., 2016). Studies in the last decades have demonstrated that epigenetic mechanisms control key aspects of cell division, cell growth, and cell fate commitment. Furthermore, significant advances have shown that several functions of the central nervous system (CNS) are mainly controlled by the dynamic roles of epigenetic modifications (Borrelli et al., 2008; Mehler, 2008; Hirabayashi and Gotoh, 2010). In addition, transcription can also be controlled through a direct mechanism that involves DNA regulatory elements (Kellis et al., 2014).

There are several types of non-coding DNA regulatory elements including promoters, enhancers, silencers, and insulators. Enhancers are presumed to be the most numerically prevalent regulatory elements. Evidence has demonstrated that distant enhancers can activate transcription by physically contacting promoters through the formation of chromatin loops. Furthermore, enhancer activity is highly dynamic and tissue-specific, with a significant number of them being active in the brain (Pennacchio et al., 2013; Andersson et al., 2014; Sabari et al., 2018). During neurogenesis, enhancers are involved in the spatiotemporal specification of cell fate. Moreover, in adult neurons, enhancers have an important role in neuronal plasticity, allowing dynamic regulation of gene expression (Carullo and Day, 2019). Interestingly, enhancers are enriched in specific epigenetic modifications, for example, histone marks. The identification of putative DNA regulatory elements has been one of the objectives of large consortium studies like ENCODE (Consortium, 2012) and NIH Roadmap Epigenomics Project (Kundaje et al., 2015). ENCODE’s main goal is to assign a biochemical function to the genome by studying well-characterized human and murine cell lines (Consortium, 2012). In contrast, the NIH Roadmap Epigenomics Project has focused on characterizing the epigenomic landscape of human primary tissue to better understand how epigenetics contributes to disease (Bernstein et al., 2010).

The nervous system of higher-order mammals is composed of multiple neuronal cell types derived from neural stem/progenitor cells (NSPCs). Neurogenesis not only takes place during development but also in selected regions of the adult nervous system. Studies have demonstrated that NSPCs reside in the adult mammalian brain within two discrete niches: the subventricular zone lining the lateral ventricles and the subgranular zone of the dentate gyrus in the hippocampus (Bonaguidi et al., 2011; Ming and Song, 2011). Constitutive adult neurogenesis takes place in neurogenic regions; however, it can also be induced in non-canonical sites after injury (Gould, 2007; Feliciano et al., 2015). In adults, the generation of new neurons is a finely-tuned process that can be triggered by a variety of signals including stimulation induced by locally released neurotransmitters, molecules that are secreted by other brain cells, and systemic factors that can cross the blood-brain barrier (Lee et al., 2002; Kohman and Rhodes, 2013; Triviño-Paredes et al., 2016). Adult neurogenesis is necessary for brain homeostasis and function, for example, learning and memory (Gallegos et al., 2018). Thus, NSPCs contribute to brain plasticity throughout life (Kempermann and Gage, 1999). These cells undergo coordinated changes in gene expression to adequately differentiate into neurons. Moreover, these changes are dynamically controlled by epigenetic mechanisms and regulatory DNA elements that define the temporal and spatial expression of key drivers of neurogenesis (Hirabayashi and Gotoh, 2010; Ma et al., 2010). Epigenetic characterization and active enhancer identification during neurogenesis have offered insights into brain function, and dysfunction contributing to disease (Carullo and Day, 2019).

In this review, we describe important epigenetic mechanisms involved in neurogenesis, namely, enhancers, DNA methylation, histone modifications, and chromatin architecture. We focus mainly on enhancers and describe their main features and regulatory mechanisms. We also describe experimental methods used to identify putative and active enhancers, infer target genes, and elucidate their regulatory function. We performed a comprehensive literature search and discuss prominent studies in the field of neural enhancers. Finally, we discuss emerging research focused on brain-enhancer genome-wide identification, target prediction, functional validation, and risk association for neurodegenerative diseases and brain disorders.

## Enhancers

**FIGURE 1:**
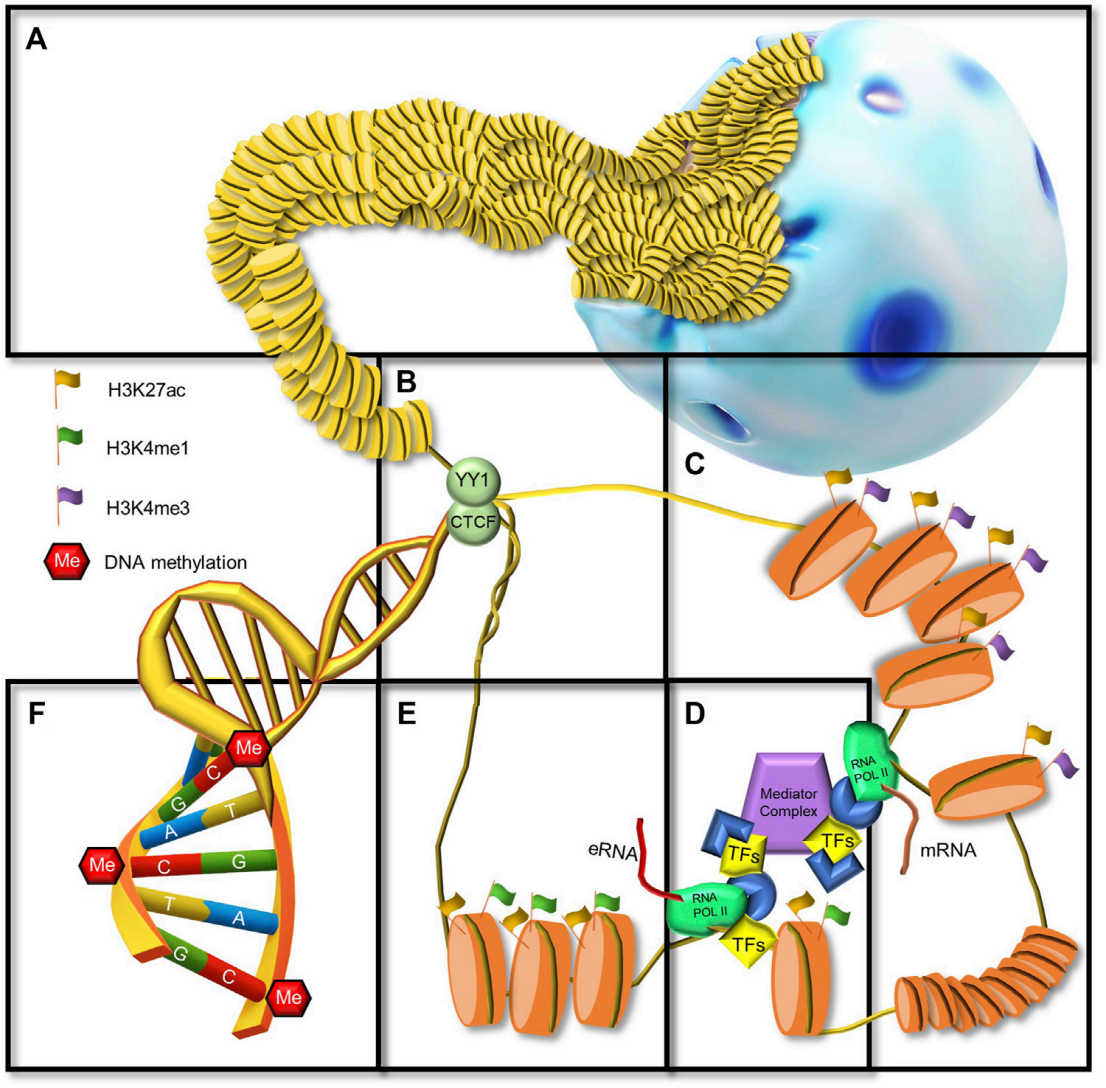
Chromatin landscape from an enhancer perspective. **(A)** Cell nucleus and densely packed chromatin (heterochromatin) in intergenic regions. **(B)** CTCF and YY1, architectural proteins of the chromatin at the anchor of a chromatin loop. **(C)** Promotor region immediately upstream a TSS, marked by H3K27ac and H3K4me3 posttranslational histone modifications, open chromatin, general TF binging, RNA POL II recruitment. **(D)** Mediator complex brings together an enhancer and a promoter. **(E)** An enhancer region marked by H3K27ac and H3K4me1 posttranslational histone modifications, open chromatin, TF binging, RNA POL II recruitment, and eRNA transcription. **(F)** DNA methylation in CpGs.

Enhancers are key players in orchestrating the dynamic transcription of the genome during embryonic development, homeostasis, plasticity, and disease. The regulatory function of enhancers is particularly important in the brain where they enable cell type diversity, neuronal plasticity, learning and memory formation, and behavioral adaptations to the environment (Carullo and Day, 2019). Enhancers are *cis*-regulatory regions of the DNA that serve as a docking platform for TF binding and increase the likelihood of transcription of one or more distally located genes in an orientation-independent manner (Figure 1C) (Gasperini et al., 2020). The activity of enhancers depends on 3D interactions with promoters (Figure 1D). Interestingly, genes can be regulated by one or multiple enhancers, suggesting functional redundancy (Osterwalder et al., 2018).

Typical enhancers are hundreds of base pairs in length (Li and Wunderlich, 2017) and they can act on target genes located as much as a million base pairs away (Lettice et al., 2003). Enhancer regions contain short (∼10 bps) sequence motifs that are recognized by TFs. Even though they depict low sequence conservation, their regulatory function is strongly conserved across the animal kingdom (Wong et al., 2020). Clusters of multiple enhancers (from a few to hundreds) referred to as super-enhancers play an important role driving the expression of genes that define cell identity (Hnisz et al., 2013). For example, super-enhancers were first identified in pluripotent embryonic stem cells (ESCs) as enhancer domains densely occupied by master TFs (Whyte et al., 2013). Moreover, super-enhancers are highly transcribed, and strongly occupied by the Mediator complex (Whyte et al., 2013). Super-enhancers identified in brain cells (microglia, neurons, and oligodendrocytes) were found to interact with cell type-specific genes and to harbor GWAS disease-risk variants, suggesting their role in neurodegenerative diseases (Nott et al., 2019).

Another hallmark of active enhancer regions is that they can be transcribed by RNA polymerase II (RNAP2), giving rise to noncoding RNAs called enhancer-derived RNA (eRNA) (Figure 1E) (Kim et al., 2010). Several studies have shown that eRNA-producing enhancers are more potent and this transcriptional activity precedes target gene expression (Arner et al., 2015; Heinz et al., 2015; Romanoski et al., 2015) eRNAs can be bidirectional and divergent and are mainly unspliced and non-polyadenylated (Sartorelli and Lauberth, 2020). Moreover, eRNAs interact with key molecules related to enhancer function, for example, the Mediator complex, transcriptional activators and coactivators, and epigenetic remodeling machinery (Bose et al., 2017). Functional studies have revealed that eRNAs contribute to gene regulation by modulating chromatin structure and function. For example, eRNAs contribute to the stabilization of enhancer-promoter loops (Li et al., 2013; Hsieh et al., 2014) and they can interact with histone acetyltransferase CREB-binding protein (CBP) to increase histone acetylation at enhancers (Bose et al., 2017). Researchers have demonstrated that the level of eRNA transcription at neuronal enhancers is positively correlated with the level of transcription at nearby promoters (Kim et al., 2010). Furthermore, eRNAs are pervasively transcribed from activity-dependent enhancers in neurons in response to neuronal activation, behavioral experience, and induced plasticity (Kim et al., 2010; Malik et al., 2014; Joo et al., 2016). This suggests that eRNAs are an important functional unit of enhancers.

The ENCODE Consortium labeled candidate *cis*-regulatory elements (cCREs) according to biochemical annotations (DNase-seq, H3K4me3, H3K27ac, and CTCF ChIP-seq) in human datasets, resulting in almost one million potential enhancers in one or more cell types (Consortium et al., 2020). Furthermore, data analysis also demonstrated that approximately 50% of cCREs with enhancer-like signatures were located in intergenic regions whereas 38% were intronic (Consortium et al., 2020). However, recent research suggests that intronic enhancers are enriched in specialized tissues and they regulate genes involved in tissue-specific functions (Borsari et al., 2021). Contrastingly, intergenic enhancers are common to many tissues and they potentially regulate housekeeping genes (Borsari et al., 2021). The authors also showed that the lowest rate of intronic enhancers was observed in less specialized tissues and ESCs samples (Borsari et al., 2021). These results highlight the role of enhancer location in the regulation of tissue-specific gene expression.

### Gene regulation by enhancers

A commonly accepted way in which enhancers regulate gene transcription is through their physical interaction with promoters. Imaging and genome-wide chromosome conformation capture assays (Hi-C) have demonstrated that eukaryotic genomes are organized into megabase-sized physical compartments referred to as topological associating domains (TADs) that are largely invariant across cell types (Dixon et al., 2012). TADs serve as the basic unit of chromosome folding and they bring distal DNA elements (enhancers and promoters) into proximity through the formation of loops (Rao et al., 2014). DNA looping is a consequence of the interaction between enhancer-bound transcription activators, Mediator, and promoter-bound RNAP2 (Figures 1C–E) (Kagey et al., 2010). Interestingly, the boundaries of TADs are enriched in binding motifs of TFs such as CTCF (Dixon et al., 2012). CTCF, a ubiquitously expressed zinc finger protein, has been implicated in numerous genome functions including transcription, splicing, insulation, and replication (Figure 1B) (Ong and Corces, 2014). Moreover, CTCF functions as an architectural protein that mediates interactions between distant sites in the genome and contributes to the establishment of a 3D chromatin structure (Beagan et al., 2017). Studies have revealed that in the mammalian brain CTCF plays an essential role in early neural development (Guo et al., 2012; Hirayama et al., 2012; Watson et al., 2014). For example, conditional knockdown of CTCF in early mouse embryonic stages resulted in the apoptosis of neural progenitor cells (NPCs), premature neurogenesis, and profound ablation of telencephalic structures (Watson et al., 2014).

Besides CTCF, several other factors regulate and stabilize enhancer-promoter loops within TADs, for example, the architectural protein YY1 (Beagan et al., 2017) (Figure 1B) and the Mediator/Cohesin complex. YY1 is a ubiquitously expressed TF that binds to active enhancers and promoters and forms dimers favoring DNA looping (Weintraub et al., 2017). The Mediator complex is a transcriptional coactivator that forms a complex with Cohesin, a ring-shaped multi-protein structure (Kagey et al., 2010). CTCF positions this complex, forming a highly dynamic DNA loop domain (Hansen et al., 2017). The Mediator complex can bind to multiple TFs bound to the enhancer as well as RNA polymerase II to communicate transcriptional signals (Figure 1D) (Allen and Taatjes, 2015). Other DNA-binding proteins or co-regulators involved in enhancer activation are the histone acetyltransferases CREB-binding protein (CBP) and P300 (Jin et al., 2011; Raisner et al., 2018). Recruitment of co-regulators and transcription of eRNAs are coupled with the covalent modification of histone tails in enhancer-associated nucleosomes (Bose et al., 2017). Therefore, these epigenetic modification patterns have been used for the identification and classification of epigenetic activation states (Ernst and Kellis, 2010).

### Identification and classification of enhancer regions

Comprehensive genome-wide studies have established that enhancers exist in multiple regulatory states and transition dynamically between them in response to stimuli (Roh et al., 2005; Heintzman et al., 2007; Heintzman et al., 2009). These states have been broadly classified as inactive, primed, poised, and active (Ernst and Kellis, 2010). Each enhancer state has specific histone modification patterns which have been used for its identification (Creyghton et al., 2010; Rada-Iglesias et al., 2011; Zentner et al., 2011).

Inactive enhancers are sequestered in compact chromatin and therefore lack TF binding, histone modifications, and promoter interactions. Enhancers can also exist in a primed state before activation (Calo and Wysocka, 2013). Even though primed enhancers are characterized by lying in accessible chromatin, depicting active histone modifications (H3K4me1) and binding of TFs and p300, they lack sufficient regulatory input to promote gene transcription (Bozek and Gompel, 2020). Moreover, primed enhancers lack H3K27ac modifications and they do not yield eRNA (Sharifi-Zarchi et al., 2017). Primed enhancers are associated with genes displaying modest expression levels and implicated in a broad range of biological processes (Creyghton et al., 2010; Zentner et al., 2011). These enhancers require additional cues for activation (Heinz et al., 2015).

Poised enhancers depict similar features as primed enhancers however, they have been found to lie within bivalent chromatin (Bernstein et al., 2006). Poised or bivalent chromatin domains carry histone modifications associated with both active and repressed states (H3K4me1 and H3K27me3) and they are correlated with pluripotency (Bernstein et al., 2006; Zentner et al., 2011). Thus, poised enhancers are related to developmental genes which are inactive (poised for activation) in pluripotent cells, (e.g., ESCs) and become activated during somatic differentiation by losing H3K27me3 and gaining H3K27ac modifications (Rada-Iglesias et al., 2011). The H3K27me3 repressive histone modification is mediated by Polycomb Repressive Complex 2 (PRC2) (Crispatzu et al., 2021). Moreover, researchers demonstrated that poised enhancers physically contact long-range target genes in mouse ESCs and this interaction is PRC2 dependent (Cruz-Molina et al., 2017). These results show that the topological interaction involving PRC2 is needed for the precise temporal activation of poised enhancers upon mouse ESCs differentiation into neuronal linages (Cruz-Molina et al., 2017).

Active enhancers induce strong expression of target genes and they may be identified through biochemical marks including: 1) enrichment of TF binding sites, 2) the ability to indirectly bind the transcriptional co-activator p300/CBP and the Mediator complex, 3) reside in accessible, nucleosome-depleted chromatin; assessed by H3K27ac and H3K4me1 overlapping modifications on flanking histones, 4) an interaction with promoters, and 5) an active transcription of eRNAs (Gasperini et al., 2020).

**FIGURE 2:**
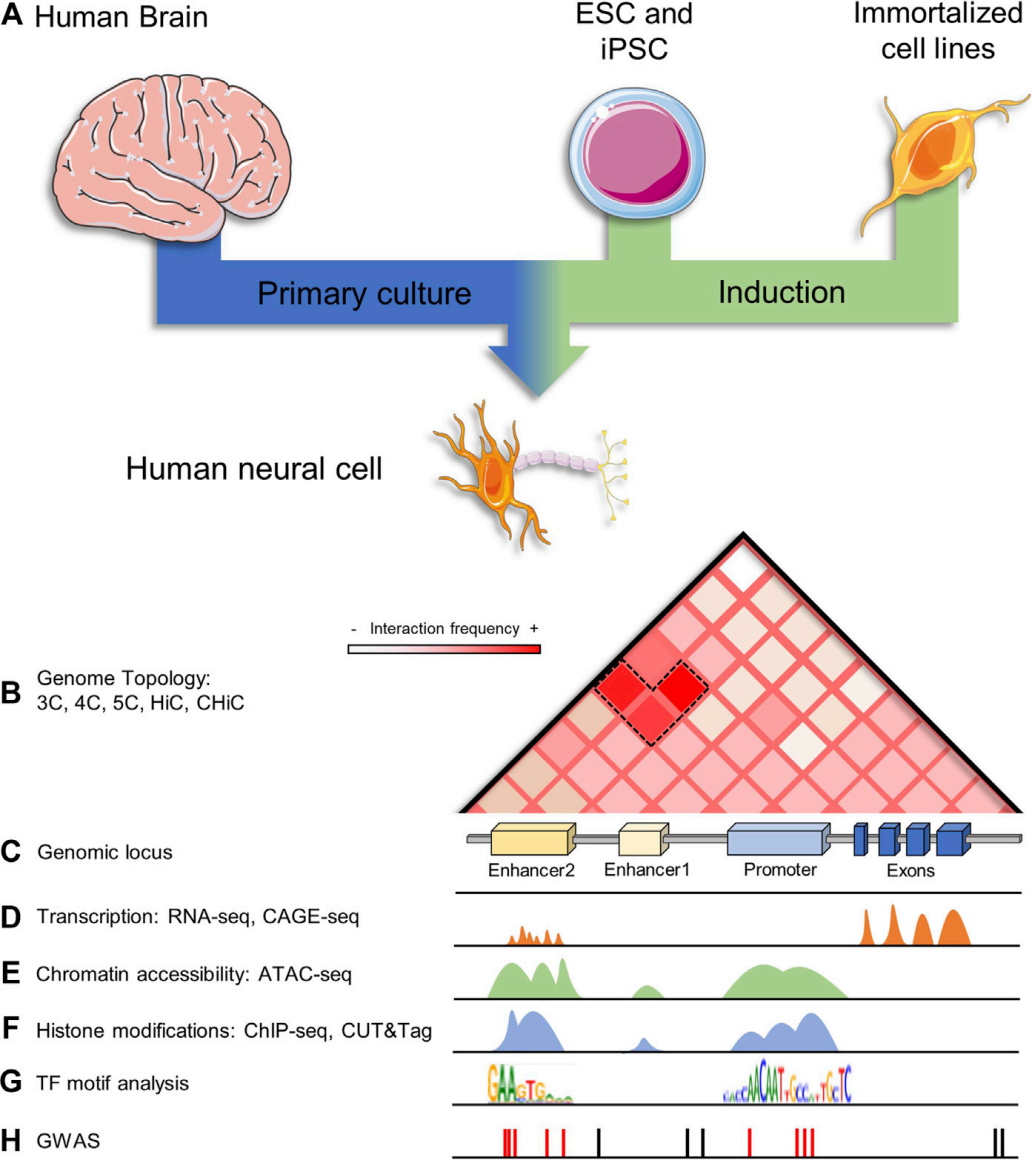
Enhancer identification and characterization in human neurons. **(A)** Human neurons for scientific study can come from different sources, like postmortem human brains at any stage (fetal or adult), they could be *in vitro* generated by ESCs and iPSCs differentiation or derived from immortalized cell lines, like LUMES and SH-SY5Y lines. **(B)** Contact matrix showing topology as measured by HiC on a genomic locus; information regarding tridimensional contacts in the genome can be also assessed by 3C, 4C, 5C, and HiC derived techniques. **(C)** A hypothetical neural genomic locus containing two enhancers and a gene promoter. **(D)** Transcription can be measured by RNA-seq and CAGE-seq, where promoters and enhancers are transcriptionally active. **(E)** Chromatin accessibility is another major feature of active regulatory elements, it can be assessed by ATAC-seq. **(F)** Enhancers and promoters can be mapped across the genome by assessing chromatin histone modifications and TF binding by ChIP-seq and CUT&Tag. **(G)** Regulatory regions are enriched in TFs binding motifs, it is possible to perform computational analysis on these elements to predict their function. **(H)** Some disease-associated SNPs lie in non-coding neural regulatory regions of the genome.

One of the most basic methods that can be employed to identify enhancers is by analyzing DNA sequence conservation as well as the enrichment of TF binding motifs (Figure 2G). However, sequence-based enhancer identification is limited since research has demonstrated that enhancers are not conserved (Schmidt et al., 2010), and not all TF motifs are known or well annotated (Figure 2C) (Lambert et al., 2018). Moreover, this method identifies ubiquitous enhancers rather than cell-specific enhancers (Fish et al., 2017; Li et al., 2019).

### Assessing enhancer activity

There are several genome-wide scale biochemical annotations used to infer enhancer activity based on their aforementioned features. For example, chromatin accessibility has been widely used to identify putative enhancers (Figure 2E). DNA accessibility can be profiled using nuclease digestion (deoxyribonuclease I hypersensitive sites sequencing, DNase-seq) (Boyle et al., 2008), micrococcal nuclease digestion combined with sequencing (MNase-seq) (Hoeijmakers and Bartfai, 2018), cross-linking and phenol-chloroform extraction to separate protein-bound and protein-free DNA fragments (formaldehyde-assisted isolation of regulatory elements sequencing (FAIRE-seq) (Giresi et al., 2007), or transposase fragmentation (assay for transposase-accessible chromatin using sequencing (ATAC-seq) (Buenrostro et al., 2013). However, since primed and poised enhancers also reside within accessible chromatin, additional information, for example, the biochemical properties of histone proteins (H3K27ac and H3K4me1) (Figure 2F), are frequently used to identify active enhancers.

Although for many years ChIP-Seq has been considered the gold standard for mapping chromatin-associated proteins, enzymatic-based methods have existed for over 20 years (Schmid et al., 2004; Johnson et al., 2007), and a new generation of these techniques has proved to be low cost, efficient and reliable, even to a greater extent than ChIP-Seq (Kuscu et al., 2021). In 2017, Skene and Henikoff developed the Cleavage Under Targets and Release Using Nuclease (CUT&RUN) for mapping histone modifications and binding of TFs in the nucleus (Skene and Henikoff, 2017; Meers et al., 2019). In this method, specific features of the chromatin are targeted with an antibody that is later bound by a micrococcal (MNase) restriction enzyme. Protein-DNA complexes in proximity to the antibody-binding site are released and used to build a library suited for massive sequencing (Skene and Henikoff, 2017). Recently, Henikoff and collages derived another antibody-directed enzymatic method: Cleavage Under Targets and Tagmentation (CUT&Tag) (Figure 2F), which in turn uses a hyperactive transposase (Tn5), *in vitro* loaded with sequencing adaptors, to release and tag protein-DNA complexes, simplifying the library preparation process and facilitating the mapping by having an increased resolution (Henikoff et al., 2020). The high performance of CUT&Tag even when used with low-cellular inputs has allowed the mapping of chromatin proteins in various cellular contexts like ESCs and it has been successfully used at a single-cell resolution level (Kaya-Okur et al., 2019).

Transcription of eRNAs has also been used as a reliable predictor of active enhancers (Figure 2D) (Wang et al., 2011; Andersson et al., 2014; Dong et al., 2018). However, since eRNAs are not readily detectable in steady-state RNA-sequencing, their identification relies on techniques that capture nascent RNA molecules. Examples of these techniques include global run-on sequencing (GRO-seq) (Core et al., 2014; Lai et al., 2015; Rahnamoun et al., 2017), precision run-on nuclear sequencing (PRO-seq) (Mahat et al., 2016), and cap analysis gene expression (CAGE) (Shiraki et al., 2003). Researchers performed a systematic identification of noncoding elements actively transcribed in dopaminergic neurons of the substantia nigra pars compacta isolated through laser-capture microdissection from 99 human post-mortem brains (Dong et al., 2018). All transcripts were ultra-deeply sequenced using ribodepleted RNAs from more than 40,000 neurons. Interestingly, authors found that nearly two-thirds of the genome was being transcribed and one-third of those coincided with enhancers defined by one or more genomic or epigenomic features (DNase I hypersensitivity sites (Thurman et al., 2012) characteristic histone modifications (Heintzman et al., 2007), CAGE-defined enhancers (Andersson et al., 2014), transcriptional coactivator p300 binding sites (Visel et al., 2009), TF “hotspots” (Yip et al., 2012), and sequence conservation (Engstrom et al., 2008; Dong et al., 2018).

Although genome-wide enhancer prediction has allowed scientists to identify putative enhancer activity in multiple cell types and stages, these methods still pose limitations. These methods represent the “operational” definition of an enhancer rather than biological validation (Gasperini et al., 2020). In this sense, although some genomic regions fulfill the properties, they might not have the biological function of enhancing gene expression (Wang et al., 2018). Also, some genomic enhancers may carry out their biological function but lack these biochemical properties. Therefore, experimental assays need to be carried out to validate the function of candidate enhancers. A common approach for testing the functional activity of thousands of candidate enhancers in a single experiment is the massively parallel reporter assay (MPRA) [reviewed in Inoue and Ahituv (2015)]. MPRAs involve cloning candidate enhancer sequences into a reporter vector where they are linked to a minimal promoter and a reporter gene (e.g., GFP, LacZ, and luciferase). Reporter gene transcripts include a barcode to identify the corresponding enhancer and these reporter vectors are transfected into cell lines or organisms. A microarray chip is programmed and barcode transcription is then normalized to the abundance of each RNA barcode to assess activity. A major limitation of MPRA is that the episomal location of plasmids eliminates the genomic context of the enhancer element. A lentiviral MPRA method has also been developed; it provides chromosomal integration and a higher correlation with ENCODE annotations (Inoue et al., 2017). High-throughput technologies that use MPRA have also been proposed, for example, Self Transcribing Active Regulatory Regions sequencing (STARR-seq) (Arnold et al., 2013). STARR-seq makes use of the ability of enhancers to work independently of their position and orientation with respect to a TSS. In this method, millions of DNA fragments are randomly generated by shearing and then they are cloned and placed downstream of a minimal promoter in a reporter gene. The resulting vector libraries are transfected into a relevant cellular context where enhancers transcribe themselves. Thus, expression abundance of each element is a quantifiable measure of its regulatory strength and can be traced down by barcoding (Arnold et al., 2013). STARR-seq was recently used to screen a library of candidate brain-specific enhancers *via* recombinant adeno-associated virus delivery to early postnatal mouse brains (Lambert et al., 2021).

### Enhancer target identification

To identify enhancer target genes, enhancer-promoter contacts may be determined through chromosome conformation capture (3C) techniques and its variants circular chromosome conformation capture (4C), and chromosome conformation capture carbon copy (5C) (Figure 2B) (Van Steensel and Dekker, 2010). Here, chromosomal contacts are fixed, DNA is sheared, and spatially close fragments are ligated, resulting in chimeric DNA molecules. After deep sequencing, long-range spatial contacts can be identified. 3C-based methods followed by sequencing (Hi-C) have allowed the identification of large-scale compartments including TADs (Dixon et al., 2012; Nora et al., 2012; De Laat and Duboule, 2013) and potential enhancer-promoter loops (Figure 2B) (Roh et al., 2005). To enrich potentially functional interactions, 3C techniques have been coupled with biochemical assays. For example, chromatin interaction analysis by paired-end tag sequencing (ChIA-PET) is a method that integrates 3C and ChIP technologies to assess protein binding (generally RNA polymerase II, CTCF, or p300) in interacting sites (Li et al., 2010; Kieffer-Kwon et al., 2013; Zhang et al., 2013). Researchers performed ChIA-PET assays on mouse NSCs and identified thousands of genes depicting long-range interactions with Sox2-bound epigenetically defined enhancers (Bertolini et al., 2019). These results confirm the importance of Sox2 as a master regulator in NSC maintenance as well as in brain development. In a modified genome conformation assay, capture Hi-C (CHi-C), researchers successfully mapped long-range promoter interactions in human cells with high resolution (Mifsud et al., 2015). Their results showed that transcriptionally active genes depicted long-range interactions with enhancer-like elements whereas inactive genes contacted elements harboring repressive marks (Mifsud et al., 2015).

In recent years, advances in microscopy have allowed the characterization of tridimensional chromatin structures and the validation of physical contact between promoters and *cis*-regulatory elements (Parteka-Tojek et al., 2022). Another way of identifying enhancer targets is through expression quantitative trait locus (eQTL) mapping of enhancer regions (Consortium, 2013). An eQTL is a genomic locus that explains a variation in transcript expression levels. eQTL studies associate genome-wide genomic and transcriptomic datasets from the same individuals to identify correlations. Variants significantly associated with differences in gene expression are considered eQTLs. Through eQTL studies, variants located within distal candidate enhancer regions can be linked to their target genes (GTEx Consortium, 2017). An important source of datasets for performing eQTL studies in human is the Genotype-Tissue Expression (GTEx) project which consists of genome-wide sequencing and gene expression profiles of different tissues from the same individual, including 14 brain regions (Consortium, 2013). Integration of eQTLs from GTEx with enhancer regions from the Roadmap Epigenomics project revealed that *trans*-eQTLs, as opposed to *cis*-eQTLs, are enriched in enhancer regions and are more cell-type-specific (GTEx Consortium, 2017). Thus, eQTL mapping is useful for identifying genomic regions responsible for tissue-specific gene expression variation. It also provides human *in vivo* validation of the effect of regulatory regions on gene transcription.

Identification of enhancer targets is crucial for comprehensive annotation of enhancer function, however, there are still numerous limitations. For example, 3C library construction requires two steps, crosslinking and proximity ligation, that are prone to introducing noises and artificial interactions (Xu et al., 2020). Furthermore, formaldehyde crosslinking suffers from preferential crosslinking (Poorey et al., 2013; Teytelman et al., 2013). Novel methods have been proposed to overcome these restrictions. Another important limitation is the resolution; typically, 3C-based methods and Hi-C yield resolutions in the order of kilobases and megabases, respectively (Figure 2B) (Jin et al., 2013). Moreover, enhancers may depict several gene targets and their association often differs depending on the technique used (Fishilevich et al., 2017), posing an additional obstacle. Also, a gene promoter for a proximal gene might act as an enhancer for a distal gene (Li et al., 2012). Finally, and most importantly, spatial proximity does not necessarily reflect regulatory function (De Laat and Duboule, 2013; Gibcus and Dekker, 2013).

An important tool for studying gene regulation is a catalogue of validated human enhancers. The GeneHancer database is a collection of reported human enhancers from different genome-wide databases: the Encyclopedia of DNA Elements (ENCODE), the Ensembl regulatory build, the functional annotation of the mammalian genome (FANTOM) project, and the VISTA Enhancer Browser (Fishilevich et al., 2017). The GeneHancer database contains information derived from 46 different cell types and enhancers are linked to genes using five methods: tissue-specific co-expression between genes and eRNAs, eQTLs from variants within enhancers, promoter-specific capture Hi-C, and TF co-expression (Fishilevich et al., 2017). Each gene-enhancer is assigned a score representing the degree of confidence. The GeneHancer database is a very good reference, however, the main goal is to have a comprehensive catalogue of functionally validated human enhancers. Due to the nature of enhancers, this database should be annotated with cell type and degree of gene activation, among other characteristics describing the biological context.

### Neural enhancers

In the last decade, high throughput sequencing techniques have allowed comprehensive mapping of regulatory elements such as enhancers in the nervous system (Nord and West, 2020). Researchers are focusing on the brain to explore the contribution of enhancers to cell fate and function and their dysregulation in disease. Among brain cells, neurons have gained most of the attention due to their dynamic transcriptional regulation required for learning, memory formation, behavioral and environmental adaptations, and stimulus-dependent induction, among others (Gallegos et al., 2018). To assess the extent of research related to neuronal enhancer activity we performed a comprehensive literature search in PubMed database. The resulting manuscripts were further filtered keeping only those published after 2017. Our search yielded 110 manuscripts which we categorized into four groups according to their research aim: enhancer regulation, enhancers and 3D chromatin conformation, enhancers involved in brain development, and disease-associated enhancers. Supplementary Table S1 lists manuscript titles and their categories. Selected prominent research studies from the first three categories are described in this subsection and projects in the category of disease-associated enhancers are described in the next section.

A straightforward way to identify cell-type-specific enhancers is using controlled and validated induction protocols. For example, in a recent study, ESCs were cultured for 72 h in a NSCs induction medium and active enhancers were evaluated at different time points (0, 3, 6, 12, 24, 48, and 72 h) using RNA-seq, ATAC-seq and H3K27ac ChIP-seq (Inoue et al., 2019). Researchers found that epigenomic changes occur in a sequential order. First, enhancer regions become accessible, then flanking histones acquire H3K27ac modifications, and finally, target genes are expressed. Furthermore, active enhancers were enriched in stage-specific TF motifs, such as NANOG, OCT4, and SOX2 in the early stages, whereas OTX2 and PAX6 were found in the late stages. A manually curated list of temporal neural putative enhancers that overlapped with ATAC-seq peaks was validated through lentiviral MPRA. Researchers concluded that 63% of these selected enhancers depicted temporal regulatory activity (Inoue et al., 2019). Similarly, Carullo and colleagues inferred eRNA transcription by combining ATAC-seq and RNA-seq from multiple rat neuronal cell cultures in two stimulation conditions (Carullo et al., 2020). These assays yielded eRNA-mRNA pairs which were specific for each population and stimulation conditions. Moreover, the authors demonstrated that eRNA transcription precedes mRNA induction and validated several candidates through CRISPR-based activation of eRNA synthesis. These findings underscore the highly dynamic and stage-specific activity of enhancers, eRNAs, and TFs involved in neural induction and stimulation. Understanding the dynamics of the regulatory network driving neural induction is also important for dissecting neurological diseases (Grove et al., 2019).

Aside from 2D cell cultures, more complex human forebrain development models followed by cell sorting techniques have also been used to identify enhancers in neural subtypes. For instance, using a 3D induction model of induced pluripotent stem cells (iPSC) into dorsal and ventral forebrains, researchers were able to capture gene-regulatory dynamics of human forebrain development and corticogenesis (Trevino et al., 2020). Briefly, neuronal and glial cells were isolated through immunopanning or FACS from forebrain organoids cultured for over 20 months. Samples were then used for ATAC-seq and RNA-seq and chromatin accessibility maps were combined with transcriptional profiles to delineate putative enhancer-gene interactions and lineage-specific TFs. Through comparisons with other epigenetic datasets, authors demonstrated that forebrain organoids recapitulated *in vivo* chromatin accessibility patterns across time (Trevino et al., 2020). Interestingly, results showed that cortical neurogenesis is characterized by chromatin state transitions and key TFs. Furthermore, genes and genetic variants associated with schizophrenia (SCZ) and autism mapped to distinct lineage-specific accessibility profiles, validating the link between disease risk and epigenetic and transcriptomic dynamic landscapes. In a similar approach, researchers used human iPSC-derived cortical organoids to study the transcriptomes and epigenomes driving cellular transitions between cortical stem cells, progenitors, and early neurons (Amiri et al., 2018). iPSCs were produced from fibroblasts isolated from human postmortem fetuses and samples of the cerebral cortex of the same fetus were used for comparative analyses. On induction days 0, 11, and 30 cells were randomly collected from organoids for RNA-seq and ChIP-seq. Functional elements (enhancers, promoters, or polycomb-repressed regions) were marked by peaks of histone marks H3K4me3, H3K27ac, and H3K27me3. Integrative analysis of transcriptome and enhancers demonstrated that enhancers could be classified as having an activating or repressive regulatory function.

Advances in single-cell isolation and sequencing technologies have allowed the assessment of cellular diversity and the identification of novel subpopulations during the development of different regions of the brain (Tepe et al., 2018; Zhong et al., 2018; Trevino et al., 2021). For example, single-cell RNA-seq of mouse neurons expressing Pitx3, a dopaminergic neuron TF, during development and adulthood revealed high transcriptomic heterogeneity (Tiklová et al., 2019), likely driven by enhancers. Genome-wide studies that have mapped accessible chromatin regions within promoters and enhancer regions have revealed that profiles of these *cis*-regulatory sites can accurately distinguish between cell types and lineages (Consortium, 2012; Roadmap Epigenomics et al., 2015). Thus, researchers are integrating transcriptomic profiles with chromatin accessibility at single-cell resolution to identify cell-type-specific regulomes. In a study by Lake and colleagues, nuclear transcriptomic and DNA accessibility maps were obtained from human adult postmortem visual cortex, frontal cortex, and cerebellum using single-nucleus droplet-based sequencing (snDrop-seq) and single-cell transposome hypersensitive site sequencing (scTHS-seq) (Lake et al., 2018). The integration of transcriptomic and epigenomic single-cell datasets allowed researchers to identify 35 subpopulations of non-neuronal and neuronal types and their related regulatory elements and TFs. Similarly, Sinnamon et al. performed single-cell ATAC-seq of the adult mouse hippocampus and identified eight cell subtypes including neuronal and glial cells (Sinnamon et al., 2019). Researchers were able to assess the accessibility of five previously identified brain enhancers and found that two of those were only accessible in neurons, two were exclusive of glial cells and one enhancer was accessible only in a subgroup of pyramidal neurons and a small set of dentate granule neurons. Inferring putative enhancers through the correlation of gene expression and chromatin accessibility is a powerful tool, however as previously described the assessment of other biochemical features is needed to elucidate activity.

Recent experiments have focused on the identification of genome-wide neural enhancers in human and murine models. Emerging evidence has demonstrated that in the adult human brain, neurons have the highest number of enhancers, compared to non-neuronal cells and there is high variability among neurons depending on the brain region and between developmental stages (Fullard et al., 2018). This region-dependent variability is not observed in glial cells. This is probably due to neurons’ highly specialized functions and complexity. Neuronal cells are unique in such a way that they are long-lived cells that must maintain the fate that was acquired during development and at the same time, they must respond to external and internal stimuli driving transcriptional changes (Gallegos et al., 2018). Enhancers play an important role in adaptation and network remodeling in the brain (Nord and West, 2020). Furthermore, these different cell types have been implicated in different neurological and psychiatric diseases, highlighting the need for assembling a catalog of active enhancers and their targets within each neural cell type as well as during developmental stages. Numerous neural enhancers have already been identified, however, there is still a long way to go for the generation of a neural subtype-specific enhancer catalog.

## DNA methylation

DNA methylation (DNAm) (Figure 1F) is one of the most extensively studied epigenetic modifications, especially in the context of cell differentiation. It plays a crucial role during mammalian development by repressing pluripotency-related genes as cells undergo differentiation (Smith and Meissner, 2013). DNAm is critically important in adult neurogenesis since it influences NSC maintenance, proliferation, fate specification, neuronal differentiation, maturation, and synaptogenesis (Jobe and Zhao, 2017). In DNAm, a methyl group is added to the fifth position of a cytosine ring (5 mC). This modification is catalyzed and maintained by the DNA methyltransferase (DNMT) family of enzymes: DNMT1, DNMT3A, and DNMT3B (Bestor, 2000), also known as “writers.” During embryonic neurogenesis, DNMTs exhibit spatial and temporal expression. DNMT localization studies using specific antibodies have reported stronger staining in neuronal cells compared to glia (Nguyen et al., 2007; Kadriu et al., 2012). DNMT1 is responsible for maintaining DNAm patterns after cell division, thus driving epigenetic inheritance (Hermann et al., 2004). On the other hand, DNMT3A and DNMT3B are involved in *de novo* methylation (Okano et al., 1998; Gowher et al., 2005).

Generally, DNAm at the promoter region is associated with gene silencing, whereas DNAm in the gene body is related to gene activation (Kundaje et al., 2015; Schübeler, 2015). DNAm in a gene’s regulatory region can inhibit its expression by hindering TF binding. Not surprisingly, DNAm can also regulate enhancer function (Kozlenkov et al., 2014; Heyn et al., 2016; Petell et al., 2016), DNAm can also recruit “reader” molecules known as methyl-DNA-binding proteins (MBPs) which can mediate context-specific transcriptional activation or repression, by associating with chromatin remodeling complexes (Nan et al., 1998; Schübeler, 2015). Interestingly, transcriptomic changes associated with aging have been suggested to be regulated by prominent DNAm enrichment in enhancer regulatory elements (Peters et al., 2015). DNAm generally occurs on a cysteine followed by a guanine nucleotide, or CpG site. However, studies in mouse and human brains have demonstrated that DNMTs can also methylate cytosines adjacent to non-guanine nucleotides, or CpH sites (Lister et al., 2013; Guo et al., 2014; Schultz et al., 2015). Interestingly, methylation at CpH is more abundant in neurons compared to other cell types and this mark accumulates during the establishment of neural circuits (Lister et al., 2013; Schultz et al., 2015). Methylation at neuronal CpH also represses gene expression consequently, loss of CpH methylation in enhancers may result in target gene activation. Both CpG and CpH methylation marks are depleted in active enhancers and promoters (Lister et al., 2009; Guo et al., 2014). Intriguingly, studies demonstrated a loss of CpH methylation marks at enhancers in aging neurons of older adults, this pattern was accelerated in Alzheimer’s disease (AD) neurons (Li et al., 2019). Integrative analysis suggests that methylation losses may be responsible for a pro-apoptotic reactivation of cell cyle and neurogenesis pathways in post-mitotic neurons (Li et al., 2019). These results pinpoint a link between enhancer hypomethylation, aging, and AD progression.

Unlike CpG, CpH methylation occurs *de novo* during neuronal maturation (Guo et al., 2014) and it is enriched in predicted distal enhancers (Kozlenkov et al., 2014). Price and colleagues profiled DNAm changes using whole-genome bisulfite sequencing in NeuN-sorted neurons isolated from 24 young (0–23 years of age) human samples from the dorsolateral prefrontal cortex (DLPFC) using fluorescence-activated nuclear sorting (Price et al., 2019). Intriguingly, the authors demonstrated that methylation patterns at CpG and CpH in neurons progressively diverge from a common landscape present in glial cells and bulk prenatal cortex with the most striking differences within the first 5 years of life (Price et al., 2019). These results suggest that dynamic regions of DNAm contribute to important neuronal processes (e.g., synaptogenesis) that occur during the first years of life. CpG methylation marks are more stable than CpH, thus it has been proposed that CpH methylation could function as a flexible and dynamic epigenetic modification in mammalian brains (Yao et al., 2016). The exact functional differences between these two classes of methylation marks remain elusive.

For a long time, DNA modifications were considered static: however, the discovery of Ten-eleven translocation (TET) enzymes and the development of sophisticated DNAm sequencing techniques allowed the elucidation of the cytosine demethylation pathway (Tahiliani et al., 2009; Ito et al., 2010; He et al., 2011; Ito et al., 2011). TET proteins (TET1-3), also known as “erasers,” catalyze the conversion of 5mC to 5-hydroxymethylcytosine (5 hmC) and subsequently to other derivatives 5-formylcytosine (5 fC) and 5-carboxylcytosine (5caC) (Ito et al., 2011). DNAm and demethylation are major mechanisms involved in cell fate decisions during embryonic brain development. During early gestation, NSCs self-renew through symmetric divisions. Later, during mid-gestation, NSCs switch to asymmetric divisions and differentiate only into neurons. However, in late-gestation and perinatal periods, NSCs acquire a gliogenic capacity producing astrocytes and later oligodendrocytes (Hirabayashi and Gotoh, 2005). DNAm has a role in the regulation of this neurogenesis-gliogenesis switch, critical for producing a balanced number of each cell type. For example, in mid-gestation NSCs, the promoters of astrocyte-specific genes (GFAP, S100*β*, Aqp4, and Clu) are hypermethylated (Namihira et al., 2004; Namihira et al., 2009). These modifications obstruct the binding of TFs such as STAT3 and astrocytic gene expression is suppressed. At later stages, Notch signaling induces expression of NFIA, which leads to demethylation *via* dissociation of DNMT1 allowing STAT3 binding and subsequent astrocytic-gene expression (Namihira et al., 2009).

The function of DNAm in adult neurogenesis has been an intensive research area since the brain is characterized by unique and high levels of CpH and hydroxymethylation (Kinde et al., 2015). Epigenomes of purified neurons have been profiled through chromatin accessibility and DNAm signatures (Mo et al., 2015). Interestingly, mature neurons were characterized by regions of hypermethylated DNA surrounding genes that are critical in neurodevelopment, such as Neurog2 (Mo et al., 2015). These findings suggest that mature neurons maintain traces of their progenitor development expression in their methylation patterns. However, specific DNAm signatures have been found in different brain regions (cerebral cortex, cerebellum, and pons) (Ladd-Acosta et al., 2007; Rizzardi et al., 2019) as well as within the same region (e.g., hippocampus) (Brown et al., 2008; Luo et al., 2017) suggesting an important role of these modifications in functional specialization. Moreover, cell-type-specific chromatin accessibility and DNAm profiles have been found in cis-regulatory regions (promoters and enhancers) of neurons (Stadler et al., 2011; Neph et al., 2012; Thurman et al., 2012). When comparing human neurons against non-neuronal cells, the majority of the differences in DNAm have been observed in regions located distally from the transcription start site (TSS), most likely enhancers (Kozlenkov et al., 2014), confirming the cell-type-specificity of enhancers and DNAm. Recent research identified enhancers as hotspots of DNA damage in human post-mitotic neurons (Wu et al., 2021). Authors developed a method to map sites of DNA repair synthesis by sequencing (synthesis associated with repair sequencing, SAR-seq) and they identified high levels of DNA single-strand breaks in post-mitotic neurons within enhancers near CpG sites and demethylated DNA. These results underscore the importance of DNAm in enhancers.

High throughput sequencing and single-cell sorting technologies have enabled researchers to further address the DNA regulatory landscape of the brain. Recently, Liu et al. (2021) profiled more than 100 K nuclei from 45 regions of the mouse cortex, hippocampus, striatum, pallidum, and olfactory bulb. Authors found that patterns of DNAm of excitatory neurons varied continuously along spatial locations (Liu et al., 2021). This amazing neuronal diversity is essential for mammalian brain function and allows neurons to interact, forming intricate networks that govern thought, emotion, and behavior. Consequently, dysregulation of neuronal enhancer methylation patterns is associated with neurological diseases.

## Histone modifications

Eukaryotic DNA is tightly wound around octamers of four essential histone proteins (H2A, H2B, H3, and H4) conforming chromatin. Histone sequences are highly conserved, however, their tails extend beyond the ring of DNA and are susceptible to post-translational modifications (Bowman and Poirier, 2015). These modifications define the accessibility of TFs and epigenetic modifiers to specific chromatin regions, thus modulating transcription (Bannister and Kouzarides, 2011). Furthermore, histone modifications can recruit chromatin remodeling enzymes that interact with DNA modifications (Bannister and Kouzarides, 2011). The most studied histone modifications involved in neurogenesis are methylation and acetylation of lysine residues (Ma et al., 2010).

Histone acetylation is a highly dynamic and reversible epigenetic modification. It is regulated by two protein families with antagonistic action; histone acetyltransferases (HATs) and histone deacetylases (HDACs). The addition of an acetyl group by HATs to histone H3 or H4 neutralizes the positive charge of the lysine residue, making the interaction between histones and DNA weaker, leading to transcriptional activation in most cases. Conversely, histone deacetylation increases chromatin compaction, hence HDACs have a repressive function on gene expression by removing histone acetylation. In a study performed *in vitro* using adult rat hippocampal NPCs, researchers found that an HDAC inhibitor was able to induce neuronal differentiation (Hsieh et al., 2004). These results demonstrate that deacetylated histones, maintained by HDACs, silence the expression of important neurogenic TFs, for example, NeuroD1 (Hsieh et al., 2004). In another study, researchers used transgenic mice and found that the CBP, a transcriptional coactivator with HAT activity, plays a critical role in the formation of long-term memory related to its acetylation function (Korzus et al., 2004).

Acetylation of lysine 27 in Histone H3 (H3K27ac) (Figures 1C–E) is a highly dynamic modification catalyzed by CBP. This modification is of particular interest because it distinguishes active from inactive or poised enhancers, containing only the methylation of lysine 4 at histone 3 (H3K4me1) (Creyghton et al., 2010). Moreover, it has also been demonstrated that H3K27ac plays an active role in controlling cell identity (Lavarone et al., 2019). However, in a recent study, Zhang et al. (2020) demonstrated that depletion of H3K27ac did not affect enhancer activity in mouse ESCs. Authors suggest that maintenance of enhancer activity does not only depend on H3K27ac, instead this modification most likely works in combination with other acetylation events (Zhang et al., 2020).

A total of 18 mammalian HDACs modulate histone deacetylation and they depict tissue specificity. HDAC1 and HDAC2 are upregulated in neocortical intermediate progenitors (IPs) in the SVZ of the developing brain and they regulate the spatial positioning of these progenitors by targeting Neurogenin2 (Tang et al., 2019). In the adult brain, subsets of NSCs and progenitors switch between expressing HDAC1 and HDAC2 as they undergo neurogenesis in the SVZ and the SGZ of the dentate gyrus (Foti et al., 2013). HDAC1 and HDAC2 were found highly expressed in glial cells and post-mitotic immature neurons, respectively (Macdonald and Roskams, 2008). However, further experiments are needed to elucidate the specific time, location, and cell subpopulations that are expressing each HDAC.

Another important histone modification is methylation. Histone methylation is more stable, due to its slow turnover. This modification can occur on basic residues (e.g., lysine and arginine) and it can consist of multiple methylations at a single amino acid (Greer and Shi, 2012). Unlike acetylation, which generally correlates with transcriptional activation, methylation at lysine residues can result in both activation or repression, depending on the modification site and the number of methyl groups added (Zhang and Reinberg, 2001). For example, H3K4me3 (Figure 1C) is usually accompanied by active transcription, while H3K27me3 has a repressive effect (Yao et al., 2016). In neuroepithelial cells, these two histone modifications with opposing functions (H3K27me3 and H3K4me3) have been found to coexist in the TSS of genes considered “bivalent” (Vastenhouw and Schier, 2012; Albert et al., 2017). Bivalent genes are abundant in stem cells and they are involved in cell fate commitment since they are poised (ready) to be expressed upon differentiation (Bernstein et al., 2006; Albert et al., 2017). Proteins with a function in histone methylation (“writers”) are referred to as histone methyltransferases (HMTs) and histone demethylation (“erasers”) as histone demethylases (DMTs). Interestingly, the number of existing HMTs is higher than the number of other histone modification writers, suggesting a complex regulation of histone methylation marks (Zocchi and Sassone-Corsi, 2010). Polycomb group (PcG) repressive complex (PRC) and trithorax active complex (TRXG), proteins with known lysine methyltransferase functions, have been implicated in the regulation of neurogenesis. Enhancer of zeste homologue 2 (EZH2), the catalytic subunit of PRC2, is a lysine methyltransferase that can generate H3K27me3 (Cao et al., 2002). Contrastingly, mixed-lineage leukaemia 1 (Mll1) in TRXG generates H3K4me3 modifications, antagonizing PRCs and keeping genes active (Schuettengruber et al., 2011). The counteracting functions of these two groups of chromatin modifiers (PcG and TRXG) maintain cellular memory. For example, during neocortical development, PcG complex controls the neurogenic to astrogenic transition in neural precursor cells by epigenetically suppressing the Ngn1 locus, increasing the H3K27me3 mark (Hirabayashi et al., 2009).

As previously described, chromatin is a dynamic scaffold that can be remodeled according to external cues, to regulate transcription. One of the main chromatin remodeling mechanisms is histone modifications. These modifications not only regulate the accessibility to DNA, but also recruit remodeling enzymes that can reposition nucleosomes. The majority of chromatin is present as heterochromatin, a condensed structure that is transcriptionally inactive (Figure 1A). It is characterized by the addition of one, two, or three methyl groups to H3K9 or H3K27 (Allshire and Madhani, 2018). Contrastingly, euchromatin is a more relaxed structure containing active genes. Other euchromatin features include unmethylated DNA and high levels of histone acetylation (Quina et al., 2006). Larger sections of heterochromatin are found in fully differentiated cells compared to progenitor or stem cells (Ugarte et al., 2015), allowing ESCs to differentiate towards any intraembryonic cell type. Genes involved in differentiation and development are subject to heterochromatization, allowing stable gene expression in fully differentiated cells.

A significant proportion of neurogenesis is regulated by dynamic histone modifications and 3D chromatin architecture (Kishi and Gotoh, 2018). Therefore, studying the landscapes of histone modifications and chromatin conformation in neurogenesis is a promising research area.

## Linking regulatory variation to neurological diseases

GWAS have identified loci (Figure 2C) that harbor genetic variants (typically single-nucleotide polymorphisms or SNPs) associated with risk for complex diseases and traits (Figure 2H) (Edwards et al., 2013). Large-scale GWAS have uncovered hundreds of SNPs associated with neurological and psychiatric disorders. Intriguingly, the vast majority of disease-associated variants (typically SNPs) reside within noncoding regions and many of them are far away from the nearest known gene (Figure 2H) (Maurano et al., 2012; Schaub et al., 2012). The latter suggests that noncoding SNPs might affect disease risk by altering the regulation of target genes. Furthermore, studies have demonstrated that noncoding SNPs are enriched in enhancers defined by chromatin accessibility, TF binding, and histone marks associated with transcriptional regulatory activity (H3K27ac, H3K4me1, and HeK4me3) as previously described (Maurano et al., 2012; Schaub et al., 2012). Thus, given that enhancers are highly cell-type-specific, researchers are combining cell-type-specific activity with GWAS data to identify disease-relevant cell types (Finucane et al., 2015; Skene and Henikoff, 2017; Finucane et al., 2018; Li et al., 2018).

As discussed earlier, neurons and neural precursors are among the cell types with more specific enhancer regulatory sequences (Andersson et al., 2014). Furthermore, dysregulation of these elements at the epigenetic or genetic level has been associated with predisposition to complex human neurological conditions, for example, SCZ, AD, and Parkinson’s Disease (PD) (Li et al., 2018; Rajarajan et al., 2018; Corces et al., 2020). Thus, cumulative research is aiming at understanding the role of these noncoding SNPs (particularly in enhancers) in the predisposition of neurological diseases. In this section, we describe prominent research using human brain cells to link cell-type-specific enhancers, SNPs, and their potential roles in disrupting enhancer-promoter interactions. Selected studies and their characteristics are listed in Table 1.

**TABLE 1:** List of prominent research performed using human brain cells to address the enrichment of GWAS SNPs in cell-type-specific enhancers depicting interactions with target genes relevant in neuropsychiatric diseases. For each study, important characteristics are described. ASD, autism spectrum disorder; ADHD, attention-deficit hyperactive disorder; SCZ, schizophrenia; MDD, major depressive disorder; BD, bipolar disorder; AD, Alzheimer’s disease; PD, Parkinson’s disease; IQ, intelligence quotient; HBA1C, hemoglobin A1C; ALS, amyotrophic lateral sclerosis; MS, multiple sclerosis; EPL, epilepsy; FTD, frontotemporal dementia; MP, mental process; UD, unipolar depression.

Samples	Assays performed	GWAS SNPs queried	Disease-related SNPs identified in cell-type specific regulatory regions	Major findings	References
Human iPSC–derived neural progenitor cells (NPCs) differentiated into neurons and astrocyte-like glial cells	Hi-C, RNA-Seq, ATAC-Seq	SCZ	Results showed 1,203 contacts with SCZ risk loci highly specific to neurons, 1,100 highly specific to NPCs and 425 highly specific for glia (locus in general, not specific SNPs)	Neural differentiation is associated with cell-type-specific chromatin 3D remodeling	Rajarajan et al. (2018)
60 postmortem brains ranging from embryonic development through 64 years old. 16 brain regions, both sexes, multiple ancestry, neurotypical controls	Genotyping, bulk and single-cell/nucleus RNA-seq, DNA methylation, CTCF binding sites and histone modifications (H3K27ac, H3K27me3, H3K4me3)	SCZ, AD, PD, ASK, ADHD, MDD, BD, IQ, neuroticism, height, IBD, total cholesterol levels, HBA1C	SCZ, IQ and neuroticism were enriched in the regulatory elements found exclusively in the dorso-lateral prefrontal cortex	Epigenomic remodeling drives neurodevelpmental processes through transcriptional changes which are temporal, regional, sex, and cell type-specific. Furthermore, GWAS risk loci were found enriched in specific regions, cell types and developmental stages	Li et al. (2018)
Undifferentiated and differentiated LUHMES	H3K27ac and CTCF ChIP-seq, RNA-seq	PD	Enhancers found only in differentiated LUHMES and within 400 kb of highly expressed and upregulated genes, coincide with PD risk SNPs at 11 loci harboring more than 100 PD risk SNPs	Differentiated LUHMES have significant enrichment for PD-risk SNPs in enhancers whereas undifferentiated cells do not	Pierce et al. (2018)
Microglia, neurons, and oligodendrocytes purified nuclei from resected cortical brain tissue from 6 individuals	Single-nuclei ATAC-seq, H3K27ac and H3K4me3 ChIP-seq	AD, ALS, MS, PD, EPL, ASD, MDD, BD, SCZ, ADHD, intelligence, cognitive function, risk behavior, neuroticism, insomnia	Psychiatric disorders were associated with variants in neuronal promoters and enhancers. Microglial enhancers were associated with sporadic AD variants	Cell type specificity is mainly represented by enhancers, rather than promoters. A microglial specific enhancer depicting a chromatin loop to BIN1 promoter and bearing a high score AD risk variant was deleted with CRISPR-Cas9 in pluripotent stem cells. These cells were differentiated into microglia, neurons, and astrocytes and BIN1 expression was depleted only in microglia	Nott et al. (2019)
iPSC-derived hippocampal DG-like, lower motor and excitatory neurons, and primary astrocytes	pcHi-C, ATAC-Seq, RNA-Seq	AD, ADHD, ALS, ASD, BD, EPL, FTD, MP, PD, SCZ, UD	The number of overlapping GWAS SNPs with at least one linked SNP participating in chromatin interactions is distributed as follows: 359 AD, 57 ADHD, 49 ALS, 130 ASD, 139 BD, 7 EP, 10 FTD, 1,772 MP, 88 PD, 439 SCZ, and 178 UD.	Assays performed allowed authors to compehensively annotate cell-type-specific interactions between promoters and distal regulatory regions (PIRs). PIRs are enriched in open chromatin and have motifs for TFs involved in cell fate specification and maintenance. The majority of promoters interacted with more than one PIR showing that promoters can be regulated by multiple enhancers. GWAS SNPs were analyzed and 70% of them overlap with at least one PIR in one or more cell types. Two PIRs interacting with CDK5RAP3 were validated using CRISPR deletion	Song et al. (2019)
Postmortem brains from 39 cognitively healthy individuals, 7 brain regons	Single-cell ATAC-seq and Hi-C	AD and PD	949 non-coding PD/AD related variants overlapped with peak chromatin accessibility regions and were likely implicated in enhancer-promoter interactions	Multi-omic and machine-learning approaches can be used to predict functional non-coding SNPs. Authors found novel and known non-coding AD/PD disease variants in open chromatin regions and involved in enhancer-promoter interactions. For example PD MAPT and ITIH1 loci	Corces et al. (2020)
Bioinformatic analysis of FANTOM5 enhancers and USCS primate and human genomes	Cap Analysis of Gene Expression (CAGE)	AD, PD, hypertension, type-II diabetes and osteoporosis	Results showed an enrichment of disease risk-associated-loci in ∼100 fast-evolved human neural enhancers	Human early-life fitness could have been achieved through rapid neural enhancer evolution; although this molecular changes could have led to aging-related disease vulnerability	Chen et al. (2018)
FANS-sorted NeuN+ and NeuN- cells isolated from healthy adult human prefrontal cortex	Hi-C and RNA-seq; H3K27ac ChIP-seq from previously published data (GABAergic and glutamatergic neurons)	AD, BD and SCZ	LDSC showed heritability enrichment of AD-associated SNPs in NeuN − H3K27ac peaks and SCZ and BD showed strong SNP heritability enrichment in NeuN + cells	Glial enhancer regions are implicated in AD while heritability for neuropsychiatric disease is harbored in neuronal enhancer regions	Hu et al. (2021)
Cerebral cortex of human fetal tissue at post conception day 20 and 21; FACS-sorted neuronal and non-neuronal cells derived from hIPs-derived cortical and subpallial organoids after long-term differentiation	RNA-seq and ATAC-seq	AD, SCZ, ADHD, EPL, and major depressive disorder	AD-, major depressive disorder-, and epilepsy-related SNPs were not enriched in any cluster. SCZ risk was enriched on mature glia, pallial neuron, late neuron, and constitutive clusters. ASD SNPs were enriched in glial progenitor and late neuron clusters. ADHD, SCZ, and ASD SNPs were enriched in subpallial spheroid peaks	Around 81% of ASD-associated genes show variable expression among cell types and developmental stage; 54% of those can be linked to an enhancer. ASD risk can be mapped to mid- and late- stage neurons and glial progenitor cell	Trevino et al. (2020)
Postmortem adult prefrontal cortex of 388 healthy controls and 351 individuals with BP or SCZ; bulk tissue of NeuN + FANS-sorted cells	H3K4me3 and H3K27ac ChIP-seq and Hi-C	SCZ and BP	Integration of ChIP-seq and Hi-C led to discovery of dysregulated histone peaks within dysregulated cis-regulatory domains associated with SCZ and BP. LDSC heritability risk was higher in hyper-acetylated peaks within these domains	Hyper-acetylated regions within cis-regulatory domains related to SCZ and BP are enriched in excitatory neurons. Many of these regions overlap with previously annotated fetal brain regulatory regions, suggesting that BP and SCZ pathology begins in neurodevelopmental stages	Girdhal et al. (2022)

One of the most prevalent human neurodegenerative conditions is PD. This neurological disorder is characterized by the selective degeneration of dopaminergic neurons within the substantia nigra [https://doi.org/10.1038/nrdp.2017.13]. To correlate PD GWAS risk loci with enhancer regulatory sequences, researchers performed genome-wide histone H3K27ac, CTCF occupancy, and RNA-seq assays using an established cell line (Lund human mesencephalic: LUHMES) as a model of human substantia nigra neurons (Scholz et al., 2011). Transcriptional profiles and enhancer regions were obtained from two conditions: undifferentiated and differentiated into functional dopaminergic neurons. Importantly, risk enhancers, defined as H3K27ac peaks containing PD-risk SNPs, were enriched in differentiated LUHMES cells only, suggesting that PD processes are active only in the differentiated condition (Pierce et al., 2018).

It has been demonstrated that enhancer activity is dynamic and may respond to extracellular stimuli. Thus, using an established cell line may bias the assessment of *in vivo* enhancer activity. Therefore, approaches using postmortem brains or tissue from resection surgeries have also been proposed. For instance, a recent study by Corces et al. (Corces et al., 2020) developed a cell-type-specific atlas of chromatin accessibility using bulk and single-cell ATAC-Seq on seven adult human postmortem brain regions from cognitively healthy individuals. Furthermore, chromatin accessibility was combined with Hi-C to identify enhancers and promoters, and their target genes. These results were combined with GWAS gene variants associated with PD and AD to determine whether variants affected enhancer-promoter interactions or TF binding (Corces et al., 2020). Researchers compiled non-coding PD/AD variants and found that many of them overlapped chromatin accessibility peaks and were likely involved in enhancer-promoter interactions. This analysis yielded dozens of functional SNPs and their gene targets in specific cell types. Furthermore, multiple neuron-specific putative regulatory elements were found. Overall, this study highlights the importance of integrating comprehensive multi-omic datasets to demonstrate the effect of noncoding variants on epigenomic regulation in specific cell types and their contribution to neurodegenerative diseases.

Another prominent research that used samples from human postmortem brains (Figure 2A) to study neurodevelopment was performed by Li and colleagues (Li et al., 2018). Authors generated integrated genomic and epigenomic data from multiple brain regions dissected from postmortem brains with ages ranging from embryonic development to adulthood. Assays performed included genotype, bulk and single-cell/nucleus RNA-seq, DNA methylation, CTCF binding sites, and histone modifications (H3K27ac, H3K27me3, and H3K4me3). Based on histone modifications, promoter and putative enhancer activity were also assessed. A large number of putative enhancers were regionally-, temporally-, or spatiotemporally-specific, whereas most promoters (63%) were not differentially enriched between conditions. Moreover, promoter and enhancer activities were strongly correlated with gene expression. Authors also associated region-specific putative regulatory regions with GWAS data for a variety of neuropsychiatric diseases. They found that SNP heritability in SCZ, intelligence quotient, and neuroticism was enriched in the regulatory elements found exclusively in the dorsolateral prefrontal cortex. These findings demonstrate that epigenetic regulations drive neurodevelopmental processes and they are associated with the risk to develop neuropsychiatric conditions (Li et al., 2018).

Chromatin and transcripts obtained from postmortem brain cells are prone to degradation (Dong et al., 2018). Thus, samples from resected human cortical brain tissues were used by Nott et al. (2019) to characterize transcriptional regulatory elements in different brain cell types and correlate them to disease-associated SNPs. Authors demonstrated a prominently higher cell-type-specificity of active enhancer regions within cortex-derived brain cells, compared to that of promoter regions. Enrichment of GWAS genetic variants associated with psychiatric disorders and sporadic AD was found in neuronal and microglial enhancers respectively. Furthermore, through proximity ligation-assisted ChIP-seq (PLAC-seq) chromatin loops were identified connecting distal regulatory regions to active promoters in microglia, neurons, and oligodendrocytes. For each cell type, chromatin interactions were further filtered to overlap with ATAC-seq, and ChIP-seq defined histone modifications (H3K27ac and H3K4me3). Interestingly, 83% of the PLAC chromatin interactions overlapped with super-enhancer regions in each cell type, and the majority of them harbored GWAS disease risk variants suggesting that these variants may affect gene transcription through their effect on super-enhancers in a cell-type-specific manner (Nott et al., 2019). The authors demonstrated the functionality of a microglia-specific enhancer region harboring a top-scoring AD-risk variant through CRISPR/Cas9-mediated deletion. Moreover, the selected microglial enhancer depicted a PLAC-derived chromatin loop to the promoter of BIN1 protein. Removal of this enhancer regulatory region depleted the expression of BIN1 protein only in microglia, whereas no effect was observed in neurons or astrocytes. These results demonstrate that enhancers and the enhancer-promoter interactome in brain cells are cell type-specific. Furthermore, SNPs in enhancers may disrupt this interactome and underlie neurodegenerative diseases.

**FIGURE 3:**
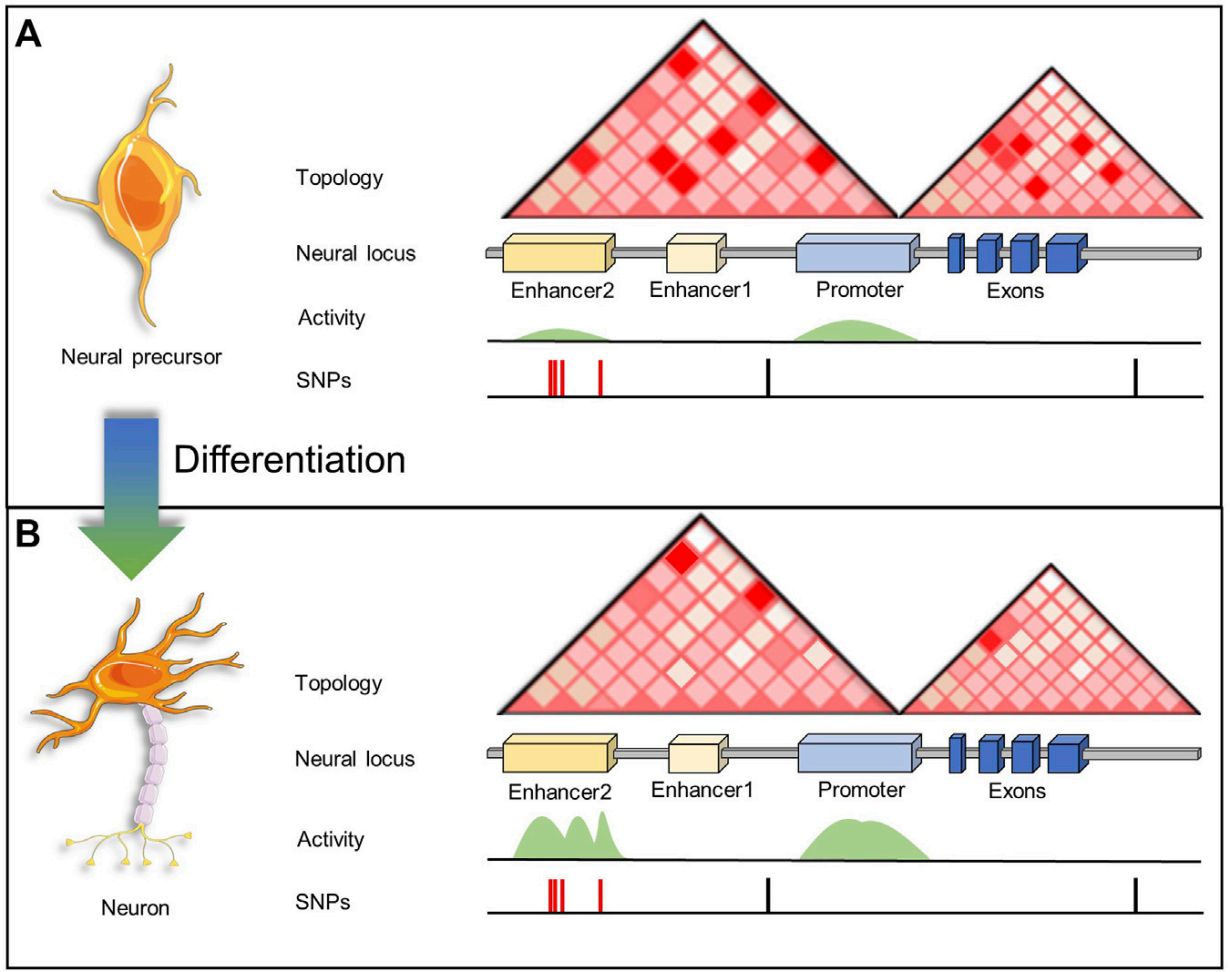
A schematic representation of enhancer activity and enhancer-promoter interactions associated to a neural locus in a NPC and a fully differentiated neuron. **(A)** In NPCs, enhancers targeting neurogenesis and neuron-related genes are interacting with promoters, however since enhancers are inactive (primed or poised) genes are not transcribed. Thus, SNPs within these enhancer regions have no effect on gene transcription. **(B)** After neuronal cell fate specification, neurogenesis and neuron-related genes are transcribed when enhancers become active. However, SNPs within active enhancers may have an effect disrupting the binding of TFs and coactivators.

Since the availability of fresh human brain tissue is limited, another approach to study disease-related SNPs in enhancer regulatory regions is using iPSCs (Figure 2A). For example, Rajarajan and colleagues studied chromatin 3D remodeling during neurogenesis generating Hi-C data from human iPSC-derived NPCs, excitatory neurons, and astrocytic cells (Rajarajan et al., 2018). The total number of chromosomal loops in neurons was 40%–50% less when compared to glial cells and NPCs (Figures 3A,B), even though chromatin accessibility profiles were very similar. Additionally, neurons depicted a greater proportion of long-range (>100 kb) loops compared to NPCs or glia. Although there are many changes in terms of loops, the overall TAD structures remained the same between brain cell types. The authors also evaluated chromatin accessibility by ATAC-seq, finding only small changes between NPCs and neurons. Thus, concluding that the 3D structural changes are not due to accessibility changes at least in the transition from NPCs to neurons. Researchers also analyzed 3D contacts in the context of SCZ-associated risk loci. Results confirmed contacts between regulatory elements and predicted target genes (based on eQTLs) in eight SCZ risk loci. There were significant 3D conformational changes across the different cell types (NPCs, neurons, and glia) in SCZ-associated loci, like the *PCDH* locus. Overall, researchers found that cell-type-specific chromosomal contacts anchored in SCZ risk sequences affected target gene expression (Rajarajan et al., 2018).

A similar study performed by Song et al. (2019). used three iPSC-derived neural linages (excitatory neurons, hippocampal dentate gyrus-like neurons, and lower motor neurons) and human GFP + astrocyte primary cultures to identify *cis* interactions between promoters and distal promoter-interacting regions (PIRs) to identify disease causal variants. For each cell type, researchers performed promoter capture Hi-C (pcHiC) and ATAC-seq genomic data coupled with RNA-seq. Results demonstrated that the majority of promoters interacted with more than one PIR showing that promoters can be regulated by multiple enhancers. Moreover, 20% of accessible PIRs in each lineage were cell-type-specific. Furthermore, GO analysis of genes contacting these cell-type-specific PIRs yielded neural-associated terms and immune functions using data from neuronal subtypes and astrocytes respectively. Using public datasets, the authors observed that PIRs are highly enriched for active chromatin features (including enhancers) inferred by ChromHMM in matched human brain tissues. PIRs also depict enrichment of H3K27ac modifications and CTCF binding sites. Additionally, neuronal subtypes depicted motif enrichment of known neuronal fate commitment TFs within PIRs. The authors validated the functional implication of PIRs in gene expression using CRISPR-Cas9 and CRISPRi. They later associated their PIRs with GWAS data for a variety of neuropsychiatric diseases, including AD and PD. A total of 6,396 unique GWAS SNPs were analyzed and 70% of them overlap with at least one PIR in one or more cell types. Autism spectrum disorder, mental process, and SCZ SNPs were enriched in PIRs across all cell types. Unipolar depression SNPs were only enriched in excitatory and hippocampal neurons, and AD, attention deficit hyperactive disorder, and bipolar disorder SNPs were enriched in motor neurons. PD SNPs displayed enrichment only in astrocyte PIRs. Accessible PIRs overlapping with neuropsychiatric SNPs contacted genes relevant to the etiology of the implicated disease (Song et al., 2019).

Figure 3 depicts the working model used by the selected research described. Briefly, by characterizing the epigenetic and transcriptomic landscapes during a differentiation process, researchers can infer the functionality of GWAS SNPs, which may alter enhancer-promoter interactions or disrupt TF binding. The latter will affect transcription only when the regulatory regions are active. Therefore, diverse layers of transcriptional regulation and epigenomics play a critical role.

The number of integrative multi-omic studies is growing as sequencing technologies become more accessible and more researchers are aiming at understanding the links between GWAS SNPs, *cis*-regulatory elements or enhancers, chromatin interactions, and regulation of transcription. Since these elements are highly cell-type-specific, researchers have used human postmortem brain regions, sorted cell subpopulations from resection tissue, iPSC-derived neuronal and non-neuronal cells, and LUHMES cell lines (Figure 2A). However, we expect to see these assays performed with single-cell resolution.

## Discussion

The human brain is a very complex organ consisting of diverse and highly interconnected cells carrying the same DNA sequence. The genome encodes the instructions for the generation and function of all cells in our body and it also harbors causes of disease susceptibility. Diversity in cell fate and function is a result of dynamic gene expression patterns that are finely controlled by regulatory elements encoded within the same DNA. Among those regulatory elements, enhancers are the most common in mammalian genomes and they are the key players in the dynamic usage of the genome during development and throughout our lifespan. Only in the brain, hundreds of thousands of enhancers have been predicted to potentially regulate spatiotemporal gene expression (Consortium et al., 2020). Mounting evidence has demonstrated that epigenetically-controlled enhancers enable neuronal differentiation, activity-dependent gene transcription, and neuroplasticity (Malik et al., 2014; Thakurela et al., 2015; Joo et al., 2016). Furthermore, an increasing number of disease-associated SNPs are being linked to enhancer function (Corradin and Scacheri, 2014; Heinz et al., 2015). Thus, neuronal enhancers are under the spotlight for studying not only brain development and function but also neurodegenerative and neuropsychiatric diseases.

High-throughput sequencing technologies have enabled genome-wide enhancer identification using biochemical signatures including chromatin accessibility, DNA methylation, histone modifications, binding of TFs and transcriptional coactivators, bidirectional transcription, and chromosomal conformation mapping (Andersson and Sandelin, 2020). Moreover, novel methods, for example, MPRA and CRISPR-Cas9, have been proposed for functional enhancer validation. These assays for studying enhancers have been performed using cell lines or cells differentiated from iPSCs, sorted from organoids, isolated from postmortem brains, or purified from resection surgeries. The existence of multiple enhancer identification and functional validation methods as well as chromatin sources poses a significant challenge in generating a consensus catalog of active enhancers in neurons. First, none of the biochemical features proposed for enhancer identification can be considered a gold standard since counterexamples have been found for each method. Moreover, the results of different assays for enhancer identification frequently disagree. For instance, studies have shown that chromatin features such as histone modifications and accessibility alone have less than a 30% chance to define a functionally active element, whereas eRNA expression is more likely to be an indicator of enhancer activity (∼70%) (Skene and Henikoff, 2017). Furthermore, researchers have demonstrated a lack of correspondence between predicted enhancers and their ability to drive expression in reporter assays (Kwasnieski et al., 2014; Inoue and Ahituv, 2015). The latter may be due to limitations in identification and validation methods but also to differences in the endogenous cellular context affecting enhancers’ dynamic nature. However, as the technologies for massively testing enhancers improve, we expect a higher number of functionally verified enhancers and their target genes, associated with cell type. This will advance our ability to predict functional enhancers from biochemical annotations, using, for example, machine learning strategies (Pliner et al., 2018; Zeng et al., 2018; Fulco et al., 2019).

Advances in novel next-generation high-throughput sequencing technologies, genome editing, GWAS, iPSC-derived cell types, and brain organoid cultures are allowing researchers to study brain development and neurodegenerative diseases with unprecedented resolution. GWAS studies have identified thousands of risk loci associated with complex brain disorders. However, the interpretation of GWAS risk loci remains challenging since most of the dysregulation is observed in noncoding regions such as enhancers. Furthermore, elucidating the effect of GWAS SNPs requires understanding cell-type-specific interactions between enhancers and promoters. Assessing this interactome is not trivial since multiple enhancers can interact with the same promoter and only some of those interactions are functional in a specific cell type and time point. Thus, the effect a SNP has on transcription is also cell-type-specific. Given that gene regulation varies substantially across cell types, future efforts should focus on characterizing the epigenomic and transcriptomic landscapes with single-cell resolution.

As discussed earlier, brain predisposition to disease in late life stages could be associated with the rapid evolution of neuronal and glial-specific enhancers, thus increasing the importance of studying these regulatory elements (Chen et al., 2018). Disregarding the challenges, combining genomics and transcriptomics with MPRA or CRISPR-based screening holds great promise for expanding our understanding of enhancer-driven regulation in specific cell types and this is likely to open new avenues for building predictive and quantitative models of enhancer activity.

## Supplementary material

Supplementary Table S1 List of papers found through a comprehensive literature search in NCBI PubMed database using the query: {[[neuron(Title/Abstract)] or [neural(Title/Abstract)] or [neuronal(Title/Abstract)]] or [brain(Title/Abstract)]} and [enhancer(Title/Abstract)] from 2017–2022. Papers were classified into five recurrent topics related to neural enhancers: disease, development, regulation, 3D chromatin conformation, and others.
